# Students’ learning experiences of three-dimensional printed models and plastinated specimens: a qualitative analysis

**DOI:** 10.1186/s12909-022-03756-2

**Published:** 2022-09-28

**Authors:** Shairah Radzi, Ramya Chandrasekaran, Zhen Kai Peh, Preman Rajalingam, Wai Yee Yeong, Sreenivasulu Reddy Mogali

**Affiliations:** 1 Lee Kong Chian School of Medicine, Nanyang Technological University Singapore, Singapore, Singapore; 2 Singapore Centre for 3D Printing, School of Mechanical and Aerospace Engineering, Nanyang Technological University Singapore, Singapore, Singapore

**Keywords:** 3D printing, Plastinated specimens, Anatomy, Medical education, Students’ perceptions, Qualitative

## Abstract

**Background:**

Traditional cadaveric dissection is declining whilst plastinated and three-dimensional printed (3DP) models are increasingly popular as substitutes to the conventional anatomy teaching and learning methods. It is unclear about the pros and cons of these new tools and how they impact students’ learning experiences of anatomy including humanistic values such as respect, care and empathy.

**Methods:**

Ninety-six students’ views were sought immediately after a randomized cross-over study. Pragmatic design was used to investigate the learning experiences of using plastinated and 3DP models of cardiac (in Phase 1, *n* = 63) and neck (in Phase 2, *n* = 33) anatomy. Inductive thematic analysis was conducted based on 278 free text comments (related to strengths, weaknesses, things to improve), and focus group (*n* = 8) transcriptions in full verbatim about learning anatomy with these tools.

**Results:**

Four themes were found: perceived authenticity, basic understanding versus complexity, attitudes towards respect and care, and multimodality and guidance.

**Conclusions:**

Overall, students perceived plastinated specimens as more real and authentic, thus perceived more respect and care than 3DP models; whereas 3DP models were easy to use and prefered for learning basic anatomy.

**Supplementary Information:**

The online version contains supplementary material available at 10.1186/s12909-022-03756-2.

## Background

Human cadaveric dissections are standard teaching and learning methods used in medical education since the seventeenth century [1, 2]. However, delivering anatomy courses through the traditional dissection mode is declining due to limited access, high maintenance of cadavers [3, 4], significant drop in contact hours for anatomy teaching [1, 5] and technological advancements [3, 6]. This opened new opportunities to investigate novel teaching methods and tools such as plastinated human specimens and three-dimensional printed (3DP) models [6–8].

These tools have their pros and cons. Plastinated specimens are dry, odourless, life-like and non-hazardous [9–11], making them ideal for teaching and engaging students to experience and appreciate anatomy. However, they are also hard and less flexible [10, 12]; thus, perceived more challenging to manoeuvre and reach deeper structures [9]. In terms of costs, plastinated specimens are usually more expensive to procure and maintain than 3DP models [6–8]. 3DP models on the other hand, allow different textures [7, 13] and colours [6, 14], and can be assigned to specific parts, which helped students identify, differentiate and remember important structures more easily, albeit perceived less realistic than the plastinated specimens.

Many studies investigated the learning outcomes/efficacy between various types of anatomy tools such as plastinated specimens, 2D images, wet prosections, Anatomage table (Anatomage Inc., San Jose, CA) and 3DP models [11, 15–21]. However, depending on the choice of learning tools used between the control and intervention groups, as well as different anatomical regions [14, 22], the results varied. For instance, the students’ learning satisfaction and their attitudes towards plastinated specimens were higher when combined with wet prosections [11, 15] and Anatomage table [20]. Similarly, the use of plastinated specimens reflected positive outcomes in the students’ objective knowledge [23, 24].

3DP models are commonly used to supplement conventional teaching and learning methods [14, 17, 21]. Loke et al., (2017) reported using 3DP models in learning congenital heart disease in pediatric residents [18]. This study found that the 3DP group reported enhanced learning satisfaction, better understanding of the tetralogy Fallot and an increased ability to manage their patients (self-efficacy) compared to the 2D image group. Similar learning satisfaction was found between learning the vascular tree anatomy and skull anatomy using 3DP models compared to the 2D images [16, 17]. These studies suggested that 3DP models were superior in the perceived learning satisfaction in students compared with 2D illustration. Studies specifically comparing multi-material 3DP models and plastinated specimens however, were limited. Mogali et al., (2021) used plastinated and their 3DP models of the cardiac and neck, and reported similar knowledge gain between the control and intervention groups [21].

Nevertheless, more evidence is needed to gain deeper insights as to why the students’ learning experiences vary with the choice of anatomy tools and for different body regions and organs [14, 22]. One interesting aspect to look out for which may influence these perceptions is humanistic values. This referred to the respect, care, empathy and compassion that students ought to have when they become doctors [25, 26]. Humanistic values are traditionally sought in cadaveric dissections as students are trained to empathize and care for the donated bodies and as such, have always held a special place for anatomy learning [27, 28]. However, this is rarely measured in plastinated and 3DP tools. Unlike closed Likert survey questions, qualitative data collection methods such as focus group discussions and open-ended survey questions allow deep insights into the free text comments of the participants to explain the impact of novel teaching tools on their learning experiences.

Therefore, this study hopes to answer how do the students’ perceptions vary when they were given established tools (plastinated) compared to its physical representation by 3D printing for learning anatomy?

### Conceptual framework

To answer the question above, students are provided opportunities to acquire, construct and share anatomy knowledge through team interactions and collaborations. This concept is well aligned with the constructivism theory, where individuals or social communities actively construct and share their own knowledge [29]. Such interactions (e.g. peer-peer; student–teacher) influence learning satisfaction [30, 31]. At the same time, students’ learning experiences can also be impacted by factors such as learning convenience, environment, teaching modalities and course contents [32]. Subsequently, these attributes can affect students’ learning and mastery of the topic of interest [33, 34]. This could relate to the pragmatism epistemology theoretical perspective, where the initial gain or formulation of one’s own experiences, intelligence and beliefs can determine the next course of action [35]. Pragmatic approaches are well-planned, are able to identify complex themes, and occur sequentially via interviews and surveys, followed by thematic analysis [36].

Cadaveric specimens are generally considered as silent mentors as they are perceived as meaningful gifts for the benefit of science and humanity, which invoked students’ respect and gratitude towards the donors [37, 38]. Previous studies reported similar or superior objective performance between the cadaveric/plastinated and 3DP groups [21, 39], it is unclear whether students hold similar learning experiences including humanistic values between the two groups. For further investigation, the pragmatism principle was used in this study [36], in which the learning experiences and characteristics of the 3DP models (colours and textures) are explored, compared with plastinated specimens by means of students’ feedback.

Subsequently, students’ perceptions may impact the educators’ decision in the selection of suitable anatomy tools based on what works and what doesn’t work for the teaching and learning of anatomy. This information may also be helpful for educators to identify the learners’ preferences and the use of appropriate anatomy tools to enhance their learning experiences.

### Study goals

This qualitative study aimed to explore what students consider important learning experiences on the use of cardiac and neck plastinated specimens compared to their 3DP models. Based on a preliminary study by Mogali et al., 2018, students perceived plastinated specimens were more life-like than the 3DP models [7]. Hence, it is hypothesized that:

Given that the plastinated specimens are generated from real cadavers, it is expected that students would perceive plastinated specimens more positively than 3DP models in terms of authencity and humanistic values.

This qualitative study is connected to two previous quantitative papers [21, 40], in that the data presented in all three studies was collected simultaneously from the same sample of student participants. The first paper demonstrated similar objective performances (test scores) between plastinated and 3DP groups [21], whereas, the second paper used factor analysis to develop a psychometrically validated instrument (four factors,19 items) that measured the educational constructs such as learning satisfaction, self-efficacy, humanistic valus and limitation of the learning tools [40]. This study explores the qualitative open-ended and focus group discussions to probe the question of what students consider important for the learning anatomy with the plastinated specimens and 3D printed models. Consequently, this study differs from the two previous papers in terms of its aim/research question, data and methods used for analysis in order to gain deep insights into the students’ qualitative feedbacks (free-text comments plus focus group discussions) regarding the use of 3DP tools compared to plastinated specimens. This implies that the current study fundamentally addresses the different research question from the preceding two articles [21, 40].

## Methods

### Context

At the author’s institution, anatomy was integrated and delivered in a systems-based curriculum such as cardiorespiratory, endocrine, musculoskeletal and others in the first two of the five-year Bachelor of Medicine and Bachelor of Surgery (MBBS) programme. Plastinated specimens, plastic models, medical imaging, and 3D virtual models were routinely used in place of cadaveric dissections or wet prosected specimens to support gross anatomy practicals. Team-based learning sessions replaced traditional didactic lectures, where the application of learned knowledge is focused. At the end of each system-based module, formative anatomy practical tests are conducted via online mode which consisted of 20 single best answers (SBA) covering gross anatomy, imaging and histology. There were total of five formative tests (three in Year 1 and two in Year 2) at the time of the experiment. The summative integrated written assessment for Years 1 and 2 has two papers, each consisted of 120 SBA. Anatomy becomes a part of these assessments and assessment blueprint determines the quantity of anatomy questions to be included.

In the efforts to improve student-specimens ratio, in-house 3DP models based on plastinated specimens were explored for teaching and learning of anatomy. This provided opportunities to uncover the educational value of novel 3DP models compared to plastinated specimens before their formal integration into the anatomy course.

### Creating 3D-printed models

**Fig. 1:**
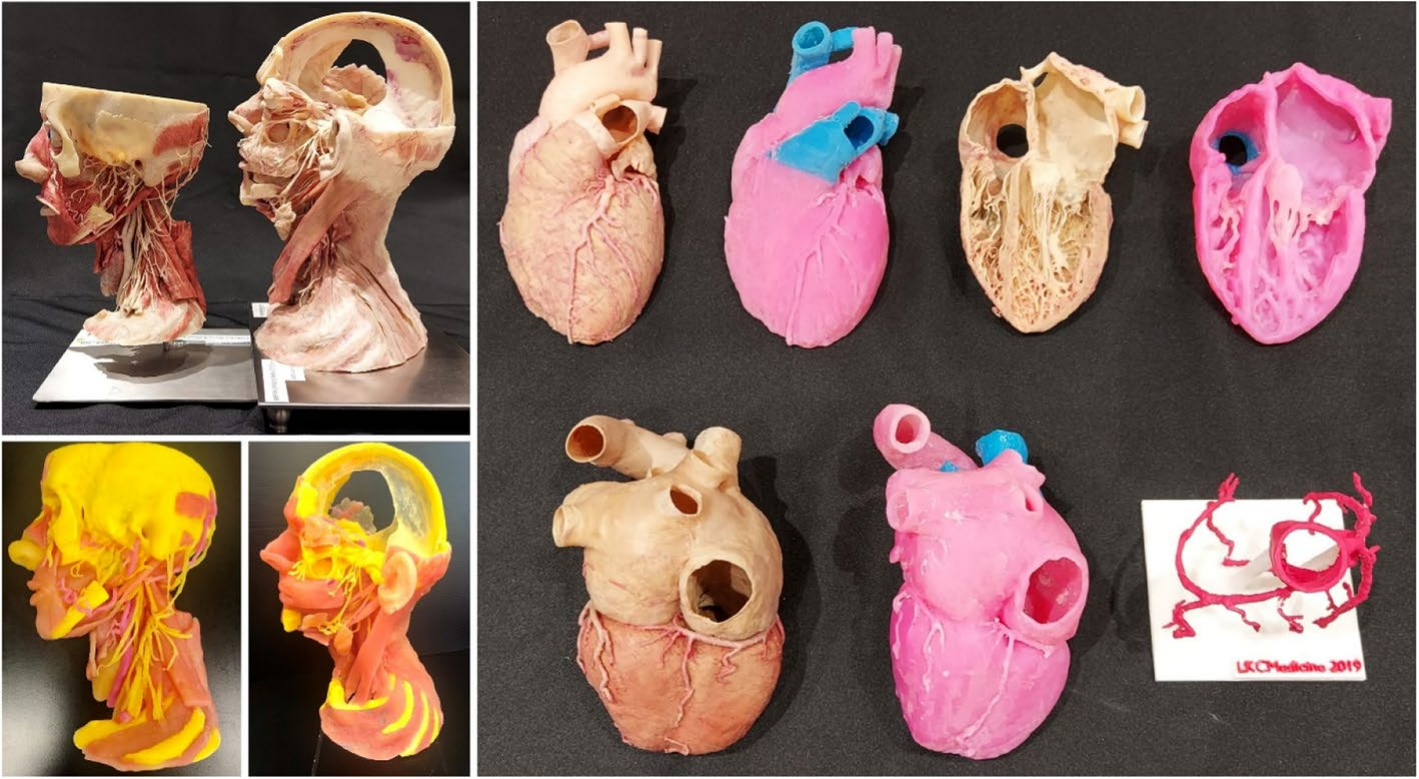
Anatomy learning tools used in this study. Left: neck; Right: cardiac plastinated and 3D-printed models

In this investigation, cardiac (one full and one cross-sectional heart) and head and neck (one full and one mid-sagittal planed head and neck) plastinated models were Computed Tomography (CT) scanned (64-slice Somatom Definition Flash CT scanner, Siemens Healthcare, Erlangen, Germany) (Fig. 1). Digital Imaging and Communications in Medicine (DICOM) images were obtained and then uploaded into a 3D slicer (versions 4.8.1 and 4.10.2, Harvard Medical School, Boston, MA) for segmentation of structure based on type such as muscles, arteries, nerves and bones. The segmented files were loaded into Materialise Magics (version 22, Materialise NV, Leuven, Belgium) to eliminate noise shells and printable model saved in STL format, which was transferred to the Objet 500 Connex3 Polyjet printer (Stratasys, Eden Prairie, MN) to produce the 3D anatomical model. Under UV light, photopolymeric resins and transparent elastomers (VeroYellow, VeroMagenta, and TangoPlus) harden layer by layer, giving each anatomical structure its own texture and color.

In addition, the ascending aorta and coronary arterial system were selected from the full heart model, and a base holder was constructed to link to the model (version 22, Materialise NV, Leuven, Belgium). The model was printed using thermoplastic polyutherane (TPU) filament on the Raise3D Pro2 (Raise3D Technologies, Irvine, CA). To reveal the model's arteries, TPU-printed support material had to be removed and vessels were painted with red acrylic.

### Study design

Year 1 LKCMedicine MBBS students (*n* = 163, 94 males and 69 females) in the academic Year 2020–2021 were sent email invitations to participate in the study as a voluntary activity. A randomized cross-over experiment was conducted in two phases, firstly with cardiac anatomy and secondly neck anatomy. There was a washout period of six weeks in between the two phases to minimize carry-over effects. In both phases, students were blinded to teaching topic and group allocation. Each group had no more than six people per team. Students who were given plastinated specimens in the first phase were instead given 3DP models in the second phase. In each phase, both groups received an introductory lecture (30 min) by a third party (senior lecturer) followed by the self-study (50 min) using the given tools and a self-guided handout.

The COREQ (COnsolidated criteria for REporting Qualitative research) Checklist was used to guide the qualitative study.

### Data collection

Students offered feedback on the study's learning materials using a survey with three open-ended questions about their strengths, weaknesses, and opportunities for development. All 96 respondents provided free-form responses. Then, eight female student volunteers (*n* = 8) took part in a focus group. The interview was conducted at the Anatomy Learning Center (where the experiments were conducted) and was moderated by Investigator #4 (PhD), a male, non-anatomy faculty member with over ten years of experience in TBL facilitation who was not involved in teaching for this research group. Students did not know the researcher’s (nor the research team’s) personal traits prior to study commencement, but the consent form informed them of the purpose of the study. Only Investigator 4 and students were present for the focus group. The same researcher described focus groups to students and inquired whether they felt comfortable participating. It was encouraged that they share their 3D-printed and plastiated learning experiences. There were six guided questions for the moderator to prompt the students to elaborate (Supplementary Material 1). Examples include discussing the aspects of the anatomy tools that help with the teaching and learning experience, and the role of empathy towards the use of such specimens. ‘How would you define your experience studying anatomy using plastinated specimens and 3D printed replicas?’ was the first interview question. All questions were left open-ended to allow users to respond freely and without preconceived areas, allowing for the discovery of new data and learning tool difficulties. Participants received neither a transcription of comments nor an analysis of results. The voluntary nature of the research avoided data saturation. The whole conversation was audio recorded for the analysis.

### Thematic analysis

The audio recordings (35 min) of focus groups were transcribed verbatim and de-identified (given a pseudonym). In addition, open-ended survey questions were collected. Focus group transcripts and survey questions were imported into a Microsoft Excel spreadsheet (Microsoft Corp., Redmond, WA) for data triangulation and merging to check for comparable or consistent study findings or for fresh discoveries [41]. This was accomplished through theoretical thematic analysis [41, 42]. Each student's free-text response was added to the total number of responses. This meant that comments with multiple sentences were counted as one. Responses with the tags nil, none, or no comments were disregarded. Three researchers (one female Research Fellow with a PhD, one female Research Associate with a M.Sc, and one male Research Assistant with a B.Eng and 1–3 years of experience in medical education research) independently and inductively coded the unstructured data. The three coders used an actual drafting board to classify their scripts on post-it notes according to similarities and discrepancies. Multiple meetings were held to arrange and group the codes through the systematic and iterative identification of patterns, which resulted in the clustering of the codes to identify sub-themes (specific or common characteristics, such as positive and negative learning tool attributes) and then the formation of overarching themes [41]. To reach a consensus, Investigator #6 (Ph.D.), who is a male with 15 years of experience in Anatomy Education, validated the final themes.

### Ethical considerations

In accordance with the Helsinki Declaration, the Nanyang Technological University Institutional review board – IRB (2019–09-024) assessed the study protocol and received the necessary permission. Participants gave informed permission and were informed of their right to withdraw at any time.

## Results

Ninety-six first year MBBS students provided full informed consent, basic demographic details such as sex and age, and declared no previous formal anatomy training. Phase 1 (cardiac) and 2 (neck anatomy) had sixty-three (33 males and 30 females) and thirty-three participants (18 males and 15 females), respectively. Their age range was 18 to 21 (Mean ± SD: 19.3 ± 0.9) years old. All 96 students responded to the survey (no dropouts), while eight female students participated in the focus group. There were 278 open-ended comments on the strengths, weaknesses and things to improve. There were no inconsistencies in the data analyzed and reporting of research findings.

### Thematic findings

**Fig. 2:**
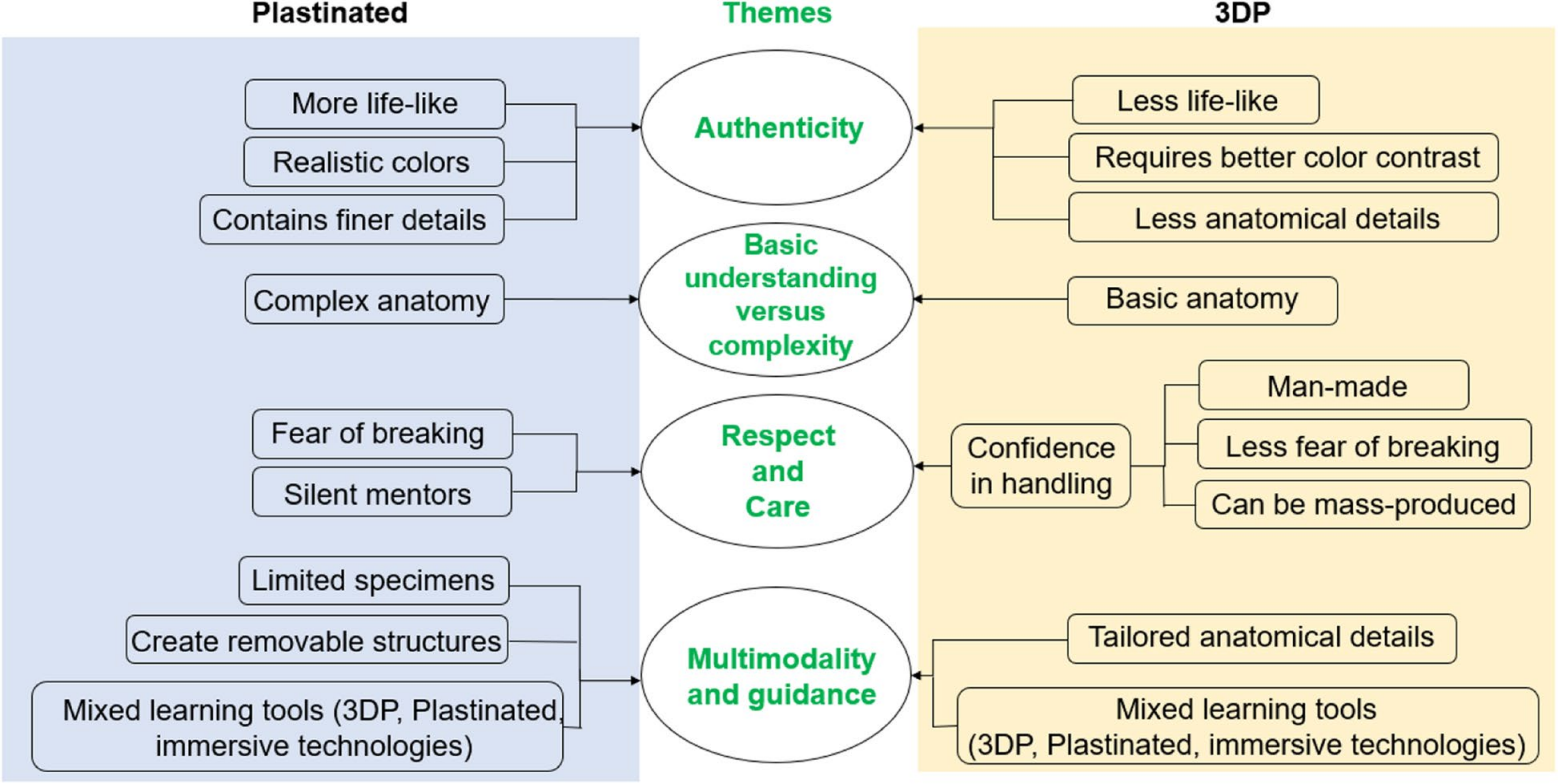
The four themes-perceived authenticity, basic understanding versus complexity, respect and care, and learning tool preferences- were based on the thematic analysis of open-ended survey questions and focus group discussion. Items in the blue and yellow boxes represented attributes of the plastinated specimens and 3DP models, respectively. 3DP = Three-dimensional printed

All the focus group discussion and survey responses generated four themes: perceived authenticity, basic understanding versus complexity, attitudes towards respect and care, and multimodality and guidance (Fig. 2). Each of these themes was described in further detail below.

#### Theme 1: The perceived authenticity

Students felt that the plastinated specimens were more life-like, had natural colours that better represent real cadavers, and had finer anatomical details compared to the 3DP models. For example, the direction of muscle fibres were more prominent in the plastinated specimens than 3DP models. This contrast was shown in the statements below.

*“…very detailed and accurate since its from a real person (Participant C17; plastinated, free text comments)”.*

*“…structures are not as precise and real…(Participant I22; 3DP, free text comments”).*

#### Theme 2: Basic understanding versus complexity

Students commented that 3DP tools were useful for learning basic anatomy and appreciation of main gross features while plastinated specimens were ideal for further expansion of their knowledge and understanding of complex anatomical structures and regions. Students perceived that they were missing valuable information when using 3DP models compared to the plastinated specimens although both tools were replicas of each other. These were explained in the statements below.

“…there were some difficulties like… minor details like the fossa ovalis… Generally using the 3D model for the heart was okay… When it came to the neck, maybe I would feel more confident learning the plastinated model (Participant PA1; 3DP, focus group discussion”).

“…could see the rough structures…To elaborate, the 3DP specimens are useful in learning, like, more, gross structures (and) larger, more identifiable things like the muscles and organs…Can be used more extensively… maybe (for) people that might not have access to plastinated specimens (Participant PA3; 3DP, focus group discussion)”.

#### Theme 3: The perceived attitudes towards respect and care

Students expressed more respect and care for plastinated specimens but also feared about breaking structures due to its fragility and inflexibility. In contrast, students were aware that 3DP models can be reproduced if damaged, hence increasing their hands-on experience.

“…we generally also do treat the plastinated specimens with more care (Participant PA2; plastinated, focus group discussion)”.

“…for the plastinated specimens it’s like this has been…something that has been preserved for so long. And if I damage it…I think we know that its like a more significant damage because it has a history (Participant PA3; plastinated, focus group discussion)”.

“3D printed models can be produced easily relatively quickly… more people can get their hands on 3D models and can facilitate learning instead of having to share a specimen (Participant I38; 3DP, free text comments)”.

“…with the 3D models we can play around a little more without too much fear of damaging them as much as if you damage the specimen…(Participant PA2; 3DP, focus group discussion)”.

#### Theme 4: Multimodality and guidance

Students quoted that the number of plastinated specimens were limited and it was difficult to access deeper structures due to its rigidity. For the 3DP models, they wished that the anatomical details can be further improved by customizing the models based on the area of interest for personalized learning. Students agreed that both the plastinated and 3DP models may be used in conjunction with other types of learning tools such as the Anatomage table to enhance the learning experience.

“Certain deep internal structures not easily visible (Participant C14; plastinated, free text comments)”.

“Perhaps the Anatomage Table and other technology would be a very useful complement (Participant C14; plastinated, free text comments)”.

“Ensure good detail in the 3D models, there could be separate models that focus on different areas and focus on different aspects, such as nerves vs. blood vessels (Participant I26; 3DP, free text comments)”.

Students also suggested incorporating faculty demonstration to explain how to utilize the models correctly or more guidance on the the images of specimens with annotations for easier learning and understanding in handouts, despite recognizing that the study was purposely designed for self-learning.

“…I do appreciate the independent learning style…perhaps more guidance could be given in the form of printed slides or some notes…(Participant C02; in general, free text comments)”.

“Content experts or having additional visual tools such as animations or videos could help us to relate better with the structures of the 3D models (Participant C38; in general, free text comments)”.

## Discussion

Medical students in their first year were asked about their learning experiences and the qualities of 3D-printed and plastinated specimens. Not unexpectedly, students found that plastinated specimens were more life-like and accurate than their 3D-printed counterparts. These results were supported by preliminary research [7]. Since plastinated specimens were made from donated bodies, they were real and authentic. Despite being a 1:1 reproduction of the plastinated specimens with similar morphometric features [8], the polymer-based 3D printed models were deemed less life-like and less realistic, particularly the finer details by the students, for example the margin of the fossa ovalis was not prominent in the cardiac 3DP model compared to its plastinate. This may be attributed to the quality of the CT images that did not clearly delineate the boundaries. Hence, it was challenging to segment this structure in the segmentation software and hence affected the 3D-printing process. This might have created skepticism on the use of 3DP tools, because they feared losing crucial learning knowledge if they did not use standard tools, such as plastinated specimens. Students interested in surgical training may see the use of actual models as essential [43]. The current findings are similar to previous studies which found that plastic models [44] and 3DP specimens lacked the precision of their real counterparts [45].

In order to increase student accessibility and subsequently learners’ satisfaction, the cost and usefulness of the tools must also be addressed. Due to their cost-effective fabrication, the results supported the use of 3DP models for acquiring anatomical knowledge [6, 21]. This was in line with prior research which demonstrated that the objective performance of plastinated and 3DP models was comparable [21]. Students felt that the 3DP models are more useful for learning fundamental anatomy concepts, organs, and prominent characteristics, while plastinated specimens were more suitable for studying intricate regions of anatomy. In addition, students advocated the use of 3DP models alongside existing cadaveric specimens and current technologies to increase students' understanding of anatomy. Multiple methods of representing the same topic, such as showing the heart anatomy utilizing cadaveric, 3D-printed, patient-scanned, and virtual 3D models. This multi-modal approach enabled students many ways of illustrating anatomy, various means to communicate what they have learned, and multiple ways to engage students’ attention [44]. The research says that realistic learning materials such as cadaveric tools can be challenging for some students in terms of cognitive load involved with studying anatomy [46]. It is essential to understand the effect of cognitive load on student learning and and apply techniques to reduce it to create a better learning environment [47, 48]. Before exposing students to the cadaveric materials, 3DP models may be a useful technique to present the fundamental and essential anatomical aspects in order to reduce cognitive load and increase learning. Additionally, students may also take the 3DP models home, integrate them with textbook and lecture materials for revision, and expand their learning of anatomy beyond the laboratory [45]. However, taking away 3DP parts is not yet practiced in the author’s institution.

In this study, plastinated specimens were regarded with more respect than 3DP reproductions. This findings was consistent with a previous study's finding that cadaveric specimens would inspire respect and sympathy as "the first patient," but artificial models would not [49]. Realistic plastinated human tissue is intimate and realistic. The use of cadaveric materials enabled students to develop humanistic and ethical ideals [50]. In addition, students' perceptions of plastinated specimens may be influenced by their growing knowledge of body donation programs and/or plastination processes. Plastinated specimens are donated bodies that can mimic the compassion, admiration, and gratitude of the learner towards the donor [10, 51]. These characteristics distinguish a humanistic caregiver and, if fostered, may aid their professional advancement by valuing their patients and evoking sympathy for them [25, 37]. This is comparable to the use of wet human dissections by silent mentors [37, 52, 53]. Due to the fact that the plastinated specimens were donated corpses, students saw them as silent mentors, which instilled respect for this novel teaching tool. Even though they were aware that the 3DP models were manufactured by machines, they enjoyed using them. Each group felt cared for and handled models with caution to preserve their integrity. Students may have been aware that the 3DP models were created from patient data for training purposes. At the authors’ institution, before the students were exposed to formal anatomy learning, an introductory anatomy session was conducted that focused on the history of anatomy followed by pledge taking by students. The main aim of the pledge was to sensitize the students with humanistic values, respect the anatomy tools and professionalism. The conjunction of anatomy tools and pledge could have assisted in inculcating the sense of care, respect and may have reminded students of future responsibilities towards the patients [54].

In terms of future improvements of the learning tools, students from both plastinated and 3DP groups related the fear of breaking off structures on their engagement and learning. During the focus group discussion, however, fear of breaking structures in plastinated specimens was particularly highlighted. This observation was supported by previous studies on plastinated specimens [9, 10]. The manipulation of structures, particularly in the neck models, were needed to explore deeper structures and comprehend 3D spatial relations. The use of haptic (touch) and visual information helped learners form a more detailed and complete 3D mental picture of the anatomical part [55]. The tactile manipulation of physical objects was found to alleviate the cognitive load and enable better understanding and retention of information [55]. It was postulated that supplementing the 3DP models with plastinated specimens may enhance students’ engagement with specimens without much apprehension of damaging the structures.

### Limitations

The participants in this study were first year medical students and provided their opinion based on two learning sessions of the cardiac and neck anatomy with plastinated and 3DP tools. These students did not have previous exposure to real human tissues or real frozen cadavers. Their perceptions, attitudes may vary with increased exposure to anatomy tools and different learning topics. Although results in this study were only based on the perceived opinions of Year 1 medical students in a single medical college, responses from 96 students were reasonable to generate meaningful new information related to the educational benefits of the plastinated specimens and 3DP replicas. Furthermore, the focus group discussion involved all females. While this may create sex bias, focus group findings were consistent with the free-text comments provided in the open-ended questions in the survey. Future studies comparing 3DP and cadaveric/plastinated materials involving different populations, cultures, and regions may be required to generalize the findings. The current study applied multi-material printing technology to create models (except the coronary arteries model). These models were found to be superior to the models produced by the fused deposition modelling (FDM) or powder-based materials in terms of spatial resolution and anatomical details [56, 57]. More research may be needed to explore student preferences and learning experiences with varying degrees of anatomy experience, and with different types of 3DP models.

## Conclusions

The 3D-printed models were perceived more suitable for learning basic anatomy, while plastinated specimens for complex anatomy. Plastinated specimens were seen as more life-like and elicited more respect and care, but the students valued the 3DP models for their capacity to provide a better hands-on experience due to their ease of handling. Educators may want to adopt a progressive strategy that uses the novel 3D-printed models as the first point of exposure in the laboratory for teaching basic anatomy, followed by highly detailed actual cadavers to benefit and maximize learning.

## Supplementary Information

**Additional file 1.** Focus group discussion guided questions (Moderator Version).

## Data Availability

Students’ feedback from the open-ended survey and text transcriptions of the focus group discussion were only accessible to the research team and not available to the public based as as per ethical approval from the university. However, relevant qualitative data to support the findings of the study have either been provided in this manuscript or as supplementary material. Detailed student responses may be made available upon reasonable request in conjunction with ethical requirements by the university, to the corresponding author Dr. Sreenivasulu Reddy Mogali (sreenivasulu.reddy@ntu.edu.sg).

## Abbreviations

3DP: 3D-printed
CT: Computed Tomography
DICOM: Digital Imaging and Communications in Medicine
FDM: Fused Deposition Modelling
LKCMedicine: Lee Kong Chian School of Medicine
MBBS: Bachelor of Medicine and Bachelor of Surgery
MOE: Ministry of Education
NTU: Nanyang Technological University Singapore
STL: Stereolithography
TBL: Team-Based Learning
TTSH: Tan Tock Seng Hospital
