# Supplementary material for: Students’ learning experiences of three-dimensional printed models and plastinated specimens: a qualitative analysis
Source: BMC Med Educ. 2022 Sep 28;22:695. doi: 10.1186/s12909-022-03756-2 (PMC9520930; doi:10.1186/s12909-022-03756-2)
Supplement: Supplementary file 1 — Additional file 1. Focus group discussion guided questions (Moderator Version). [file 12909_2022_3756_MOESM1_ESM.docx]

**FOCUS GROUP DISCUSSION GUIDED QUESTIONS (Moderator Version)**

1. To discuss what aspects of anatomy tools enhances the quality of the teaching, and the quality of the learning experience.
2. To describe about their experience of learning anatomy with plastinated specimens and 3D printed models.
3. To talk about how students would contrast the 3D printed models with the plastinated specimens.
4. To explore the students’ satisfaction and confidence in their knowledge of anatomy using plastinated specimens and 3D printed models.
5. To talk about developing or enhancing feeling of empathy from anatomy specimens
6. To explore the role of 3D printed models in future anatomical/medical education.
